# Prolonged Hemoptysis Caused by Primary Pulmonary Epithelioid Hemangioendothelioma; A Case Report and Review of the Literature

**Published:** 2014-03

**Authors:** Bita Geramizadeh, Bejan Ziyaian, Mehdi Dehghani, Kamran Tahmasebi

**Affiliations:** 1 Transplant Research Center, Department of Pathology, Nemazee Hospital, Shiraz University of Medical Sciences, Shiraz, Iran;; 2 Department of Surgery, Nemazee Hospital, Shiraz University of Medical Sciences, Shiraz, Iran;; 3 Department of Internal Medicine, Nemazee Hospital, Shiraz University of Medical Sciences, Shiraz, Iran

**Keywords:** Hemangioendothelioma, Pulmonary, Hemoptysis

## Abstract

Epithelioid hemangioendothelioma is a vascular tumor with an intermediate malignant potential. This tumor is very rare in the lung parenchyma, and most of the previously reported cases have been asymptomatic. There is no standard therapy for this tumor and prognosis in the previous reports has been variable.

Herein we report our experience with a 60-year-old woman presenting with hemoptysis and multiple lung consolidation, leading to a diagnosis of epithelioid hemangioendothelioma after surgical resection and pathological examination. After surgery and chemotherapy, the patient had an acceptable course.

## Introduction

Epithelioid hemangioendothelioma (EHE) is a rare tumor originating from the endothelial cells and is histologically characterized by an epithelioid appearance. It has been called under various names such as intravascular bronchiolar and alveolar pulmonary tumor.^1^ The most common site of this tumor is the liver.^2^ EHE has been rarely reported from the lung as a primary origin. It has an intermediate malignant potential with no standard method of treatment.^3^

Most of the reported cases have been asymptomatic and incidentally diagnosed; however, nonspecific symptoms such as chest pain, dyspnea, and productive cough have also been reported. Hemoptysis has been very rarely reported, and even extensive hemorrhage has been very rarely reported as a cause of death.^2^

Herein we report our experience with a rare case of EHE in the lung. To the best of our knowledge, fewer than 100 cases of this tumor have been reported in the lung, most of which have been asymptomatic. Our patient was a 60-year-old woman presenting with hemoptysis, which is an uncommon presentation in this tumor. 

## Case Report

A 60-year-old woman from Shiraz presented with on and off hemoptysis for 2 years and referred to Nemazee Hospital in August 2011. She also complained of left shoulder pain and mild dyspnea in the last 2 years.  

She has been a water pipe smoker for more than 20 years. 

Her medical history was unremarkable, except for total abdominal hysterectomy and bilateral salpingoophorectomy for prolapse 9 years ago.

At the time of admission, physical examination showed blood pressure of 100/60 mm Hg, pulse rate of 70/min, respiratory rate of 20/min, and temperature of 36.5°C. She had pale conjunctiva and decreased breathing sounds in the left lung. 

Laboratory examination showed WBC of 8500/mm^ 3 ^, hemoglobin of 11.2 gr/dl, and platelet of 436000/mm^ 3 ^. Additionally, prothrombin time, partial thromboplastin time, and International Normalized Ratio (INR) were all normal, and liver and renal function tests were also unremarkable.

Echocardiography was also normal. 

**Figure 1:**
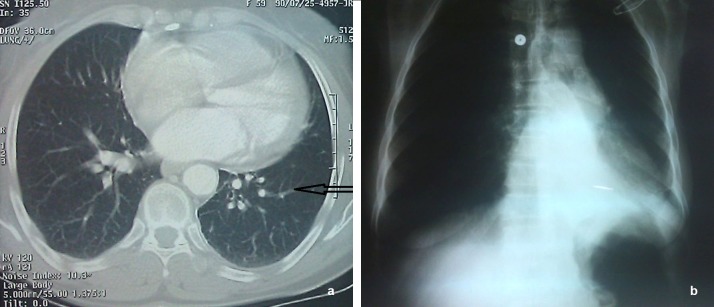
Chest X-ray, showing opacity in the left lung (a). Spiral computed tomography scan, demonstrating consolidation in the apical segment of the left lower lobe (b).

Chest X-ray showed opacity in the left lung (figure 1a). Spiral computed tomography (CT) scan showed consolidation in the apical segment of the left lower lobe (figure 1b). In the operating room, fiber-optic bronchoscopy was performed, which showed a mass in the anterior wall of the left lower lobe bronchus. Biopsy was taken. After biopsy, the patient had active bleeding, so emergency surgery was performed and a segment of the left lobe was resected.

Pathological examination on a segment of the patient's lung revealed multiple small whitish creamy micronodules, measuring 0.2 to 0.4 cm in diameter. 

**Figure 2:**
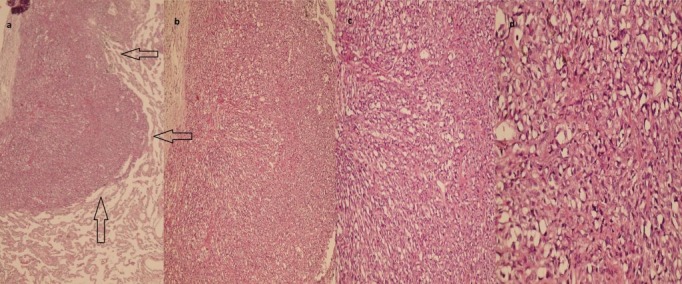
Histopathological sections, showing multiple small and large nodules of the tumoral tissue (a). High power, illustrating vacuolated cells with intracytoplasmic vacuoles (b,c,d).

**Figure 3:**
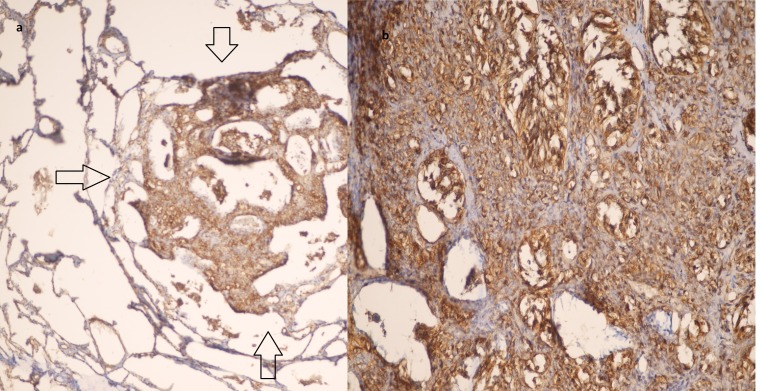
Immunohistochemical staining of the tumoral tissue, showing reactive CD31 (low power: a), high power: b)

Histopathological study of the sections from the nodules showed a tumoral tissue, extending from alveolus to alveolus. Moreover, the nuclei were bland looking and round to oval, with foci of cytoplasmic vacuolization. Mitoses were absent, and there was no necrosis (figure 2a, b, c, d). Immunohistochemistry revealed reactive CD31 (figure 3a, b) and CD34 as well as non-reactive TTF-1 and cytokeratin. The proliferative index (Ki-67) was about 10%. The diagnosis of primary EHE was made, because all the other body parts, including soft tissue, bone, and brain, were completely unremarkable.

MAID (Mesna, Doxorubicin, Ifosfamide, and Dacarbazine) regimen was started for the patient. Six months on, she is well and under follow-up.

## Discussion

EHE is a rare tumor originating from the vascular endothelial cells and characterized by epithelioid histological feature.^1^

The first description of EHE in the lung tissue was by Dail and Liebow^4^ in 1975 under the name of intravascular bronchiolar and alveolar pulmonary tumor. It was initially considered as an aggressive cancer with vessel invasion.^5^ The term "epithelioid hemangioendothelioma" was first used by Enzinger and Weiss in 1988. Now it is recognized that the true nature of this neoplasm is from the endothelial cells with a low-grade to borderline malignant potential.^6^

More than 75% of the cases of EHE are in female patients,^6^ and there is a wide age range from 7 to 83 years.^1^

Most of the reported cases have been asymptomatic and incidentally diagnosed; however, nonspecific symptoms such as chest pain, dyspnea, and productive cough have also been reported.^1^^,^^6^ Hemoptysis has been very rarely reported, and even extensive hemorrhage has been reported as a cause of death.^7^ Our patient was a 61-year-old female with a prolonged history of hemoptysis, and she underwent surgery with extensive hemorrhage.

The radiological features of the EHE in the lung can be presented as multiple pulmonary nodules, multiple pulmonary reticulonodular opacities, or diffuse infiltrative pleural thickening.^8^ The size of the nodules is commonly less than 1 cm, and they are mostly located near the medium-sized bronchial vessels.^1^ The diagnosis of EHE is always based on the pathological examination of the tissue.^6^ Nevertheless, according to the previous reports, most often the bronchial biopsies and the bronchoalveolar lavage are not informative. The diagnosis is generally made on a surgical pulmonary biopsy.^9^

Our case was also diagnosed based on the surgical specimen. It means that the bronchoalveolar lavage and biopsy were not informative, and finally the patient underwent surgery after extensive hemorrhage during bronchoscopy and biopsy.

The final diagnosis of this tumor is based on pathological examination and immunohistochemical confirmation.^10^ The pathological sections of EHE by hematoxylin and eosin staining (H&E) show a tumoral tissue, extending from alveolus to alveolus. The nuclei are bland looking and round to oval, with foci of cytoplasmic vacuolization. Mitoses are rare or absent, and necrosis is uncommon. Both vascular and lymphangitic spread has been reported.^11^ According to the available literature, the distinctive histological features of EHE are: 1) structure of the nodules; 2) presence of numerous well-formed vessels; and 3) multiple intracellular vacuoles.^4^

In immunohistochemical study, the cytoplasm of the tumor cells shows a widespread expression of CD-31. In the previous reports, reaction with CD-105 antibody has been mainly in the cytoplasm of the tumor cells in the periphery of the tumor. Reaction with D2-40 antibody has been mostly negative.^12^ In our patient, the pathological diagnosis of the tumor was straight forward and was confirmed by immunohistochemical staining. CD31 is the most specific endothelial marker, which was also positive in our patient.^1^ Negative results after staining with markers of mesothelial, epithelial, and muscular differentiation, associated with positive results for endothelial cell markers such as antibodies directed against factor VIII, CD31, or CD34, make the diagnosis definite.^4^

Because of its rarity, there is no standard treatment for this tumor. Nonetheless, in lesions that are small and limited in number, surgical resection is recommended.^1^ Different chemotherapeutical regimens have been used in the previous reported cases such as Mesna, Doxorubicin, Ifosfamide, and Dacarbazine (MAID).^13^ Other reported treatments have been Mitomycin C, 5-Fluorouracil, Cyclophosphamide, Vincristine, Tegafur or Cisplatin, Carboplatin, Etoposide, and Vinorelbine.^4^ The results of chemotherapy and tumor responsiveness have been variable. Also, radiotherapy has been reported to be ineffective.^13^ Overall, no effective and standard therapy for EHE has yet been established other than resection.^14^

Lung transplantation should be considered and evaluated in patients with vascular aggressivity with pleural hemorrhagic effusion and anemia.^15^ Our patient was treated with MAID.

EHE is a neoplasm with a highly variable clinical course, the behavior of which is intermediate between hemangioma and angiosarcoma.^15^

The overall 5-year-survival has been reported between 47-71%.^2^ In patients with asymptomatic bilateral pulmonary nodules, as well as after a curative surgical resection, regular follow-up is mandatory.^1^

Our patient has now been followed-up for about 6 months; she is currently well and symptom free.

As a conclusion, EHE is a rare vascular tumor and its occurrence in the lung is even rarer. To the best of our knowledge, there have been fewer than 100 cases in the English literature so far.^1^^-^^4^ EHE is most commonly asymptomatic, but it can rarely present with hemoptysis.

Therefore, primary pulmonary EHE should be considered as the differential diagnosis of lung masses presenting with intractable prolonged hemoptysis.
